# Leveraging social media and other online data to study animal behavior

**DOI:** 10.1371/journal.pbio.3002793

**Published:** 2024-08-29

**Authors:** Reut Vardi, Andrea Soriano-Redondo, Jorge S. Gutiérrez, Łukasz Dylewski, Zuzanna Jagiello, Peter Mikula, Oded Berger-Tal, Daniel T. Blumstein, Ivan Jarić, Valerio Sbragaglia

**Affiliations:** 1 School of Geography and the Environment, University of Oxford, Oxford, United Kingdom; 2 WildCRU, Recanati-Kaplan Centre, Department of Zoology, Oxford University, Oxford, United Kingdom; 3 School of Zoology, Tel Aviv University, Tel Aviv, Israel; 4 CEABN, Centro de Ecologia Aplicada Prof. Baeta Neves, InBIO Laboratório Associado, Instituto Superior de Agronomia, Universidade de Lisboa, Lisboa, Portugal; 5 Helsinki Lab of Interdisciplinary Conservation Science (HELICS), Department of Geosciences and Geography, University of Helsinki, Helsinki, Finland; 6 Department of Anatomy, Cell Biology and Zoology, Faculty of Sciences, University of Extremadura, Badajoz, Spain; 7 Ecology in the Anthropocene, Associated Unit CSIC-UEX, Faculty of Sciences, University of Extremadura, Badajoz, Spain; 8 Poznań University of Life Sciences, Department of Zoology, Poznań, Poland; 9 Institute of Evolutionary Biology, Faculty of Biology, Biological and Chemical Research Centre, University of Warsaw, Warsaw, Poland; 10 Faculty of Environmental Sciences, Czech University of Life Sciences Prague, Prague, Czech Republic; 11 TUM School of Life Sciences, Ecoclimatology, Technical University of Munich, Freising, Germany; 12 Institute for Advanced Study, Technical University of Munich, Garching, Germany; 13 Mitrani Department of Desert Ecology, Jacob Blaustein Institutes of Desert Research, Ben-Gurion University of the Negev, Ben-Gurion, Israel; 14 Department of Ecology and Evolutionary Biology, University of California, Los Angeles, California, United States of America; 15 Université Paris-Saclay, CNRS, AgroParisTech, Ecologie Systématique Evolution, Gif sur Yvette, France; 16 Biology Centre of the Czech Academy of Sciences, Institute of Hydrobiology, České Budějovice, Czech Republic; 17 Department of Marine Renewable Resources, Institute of Marine Science (ICM-CSIC), Barcelona, Spain

## Abstract

The widespread sharing of information on the Internet has given rise to ecological studies that use data from digital sources including digitized museum records and social media posts. Most of these studies have focused on understanding species occurrences and distributions. In this essay, we argue that data from digital sources also offer many opportunities to study animal behavior including long-term and large-scale comparisons within and between species. Following Nikko Tinbergen’s classical roadmap for behavioral investigation, we show how using videos, photos, text, and audio posted on social media and other digital platforms can shed new light on known behaviors, particularly in a changing world, and lead to the discovery of new ones.

## Abstract 2

Data from digital sources offer many opportunities to study animal behavior. Following Niko Tinbergen’s classical roadmap for behavioral investigation, this essay shows how using videos, photos, text, and audio posted online can shed new light on known behaviors and lead to the discovery of new ones.

## Introduction

Rapidly accumulating digital data offer numerous opportunities for science. With more than half of the world’s population online (https://www.itu.int/en/ITU-D/Statistics/Pages/stat/default.aspx), billions of people are generating online digital data in the form of text, images, videos, and audio uploaded to social media platforms and other websites (Box 1). Furthermore, field notes, printed books, and old news media are being increasingly digitized and made available online [1]. These vast digital knowledge repositories can provide meaningful insights into the natural world. Indeed, several emerging fields have been developed for that purpose; conservation culturomics uses digital data to inform conservation science and human–nature interactions [2], while iEcology (or passive crowdsourcing [3]) uses such data to study ecological patterns [4]. Indeed, geotagged data from multiple digital sources can complement other data to monitor distributions and occurrences of species, particularly of charismatic ones, or in and around human-dominated landscapes such as urban habitats or areas subjected to high human visitation [5,6].

**Box 1. Categories of digital data**

While using the term digital data, we distinguish between 3 major categories:

1. Digitized scientific databases, such as digitized museum records, and audio or video online libraries, that have usually been collected by researchers.
2. Citizen/community science data sets where members of the public record their nature sightings for scientific use, either for general data repositories or for specific research projects (e.g., iNaturalist and eBird).
3. Social media platforms—such as X (formerly known as Twitter), Instagram, or Google Images—where individuals upload content generated for various purposes typically not with the intention to address scientific questions yet may, nevertheless, be relevant to research.

Data from the 3 categories can differ in their collection protocols, reliability, accuracy, accompanied metadata, and data-sharing rights. While we consider the importance of data use from all 3 categories, given the novelty, extent, and challenges associated with using data from social media platforms, we focus primarily on the potential and limitations of such digital data sources.

**Fig 1:**
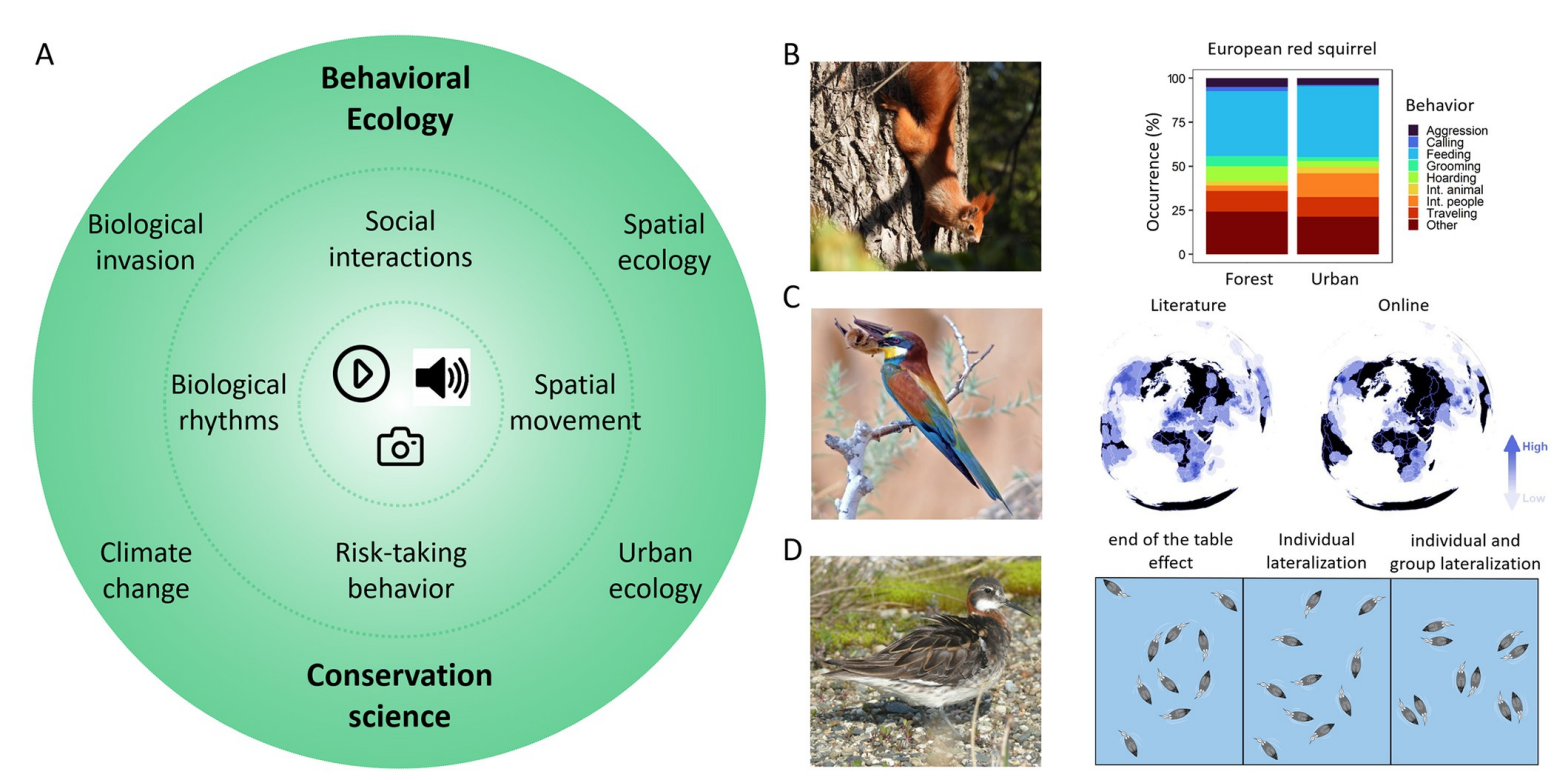
Examples of the main sources of digital data that can be used to study animal behavior. **(A)** Digital data (inner circle; photos, videos, and audio) can complement experimental and observational approaches aiming to characterize several aspects of animal behavior, such as social interactions and biological rhythms (middle circle). Applications of digital data are particularly interesting for characterizing behavioral and ecological patterns addressing several research fields (e.g., urban ecology and biological invasions) as well as tackling conservation issues (outer circle). (**B–D)** Representative examples of studies that used digital data to characterize animal behavior. (**B**) Percentage of recorded behavior in forest and urban ecosystems for the European red squirrel (*Sciurus vulgaris*) based on YouTube videos (right; adapted from [8]); photo of a red squirrel (photo credit: Peter Mikula); (**C**) Density maps showing the distribution of bat predation records by diurnal birds based on published literature (left map) and online records such as Google images, Flickr, and YouTube (right map; adapted from [10], countries borders map taken from https://public.opendatasoft.com/explore/dataset/ne_10m_admin_0_countries/map/). Example photo of a European bee-eater (*Merops apiaster*) trying to swallow a Kuhl’s pipistrelle bat (*Pipistrellus kuhlii*; photo credit: Shuki Cheled). (**D**) Wilson’s phalarope (*Phalaropus tricolor*) are shorebirds renowned for their unique spinning behavior, during which individuals rapidly spin their bodies in tight circles to upwell small prey and feed upon them. Freely available videos from YouTube, Vimeo, and Flickr have revealed that nearest neighbors of Wilson–s phalarope are more likely to spin in the same direction, thus reducing interference with each other, but not red-necked phalaropes (*Phalaropus lobatus*; photo credit: Miroslav Salek) (adapted from [11]).

Digital data can also be used to characterize animal behavior [7]. For example, Jagiello and colleagues [8] used YouTube videos to compare the occurrence of various behaviors of Eurasian red squirrels and invasive gray squirrels (*Sciurus vulgaris* and *S*. *carolinensis*) between 2 habitats. They found that calling and aggressive behaviors were more frequent in forests than in urban habitats (Fig 1). Similarly, Boydston and colleagues [9] analyzed YouTube videos to understand the structure and putative function of coyote–dog (*Canis latrans*–*C*. *familiaris*) interactions. They found evidence of intricate social behavior between the 2 species. However, YouTube is not the only platform that offers data that, while collected for other purposes, can be meaningful for behavioral ecology. Other sources may include various social media platforms (X (formerly Twitter), Facebook, Instagram, etc.), digitized scientific records, and citizen science databases (see Box 1). Such alternative sources of information may help fill important gaps in our understanding of animal behavior and shed light on how animal behavior may be influenced by humans’ actions.

**Fig 2:**
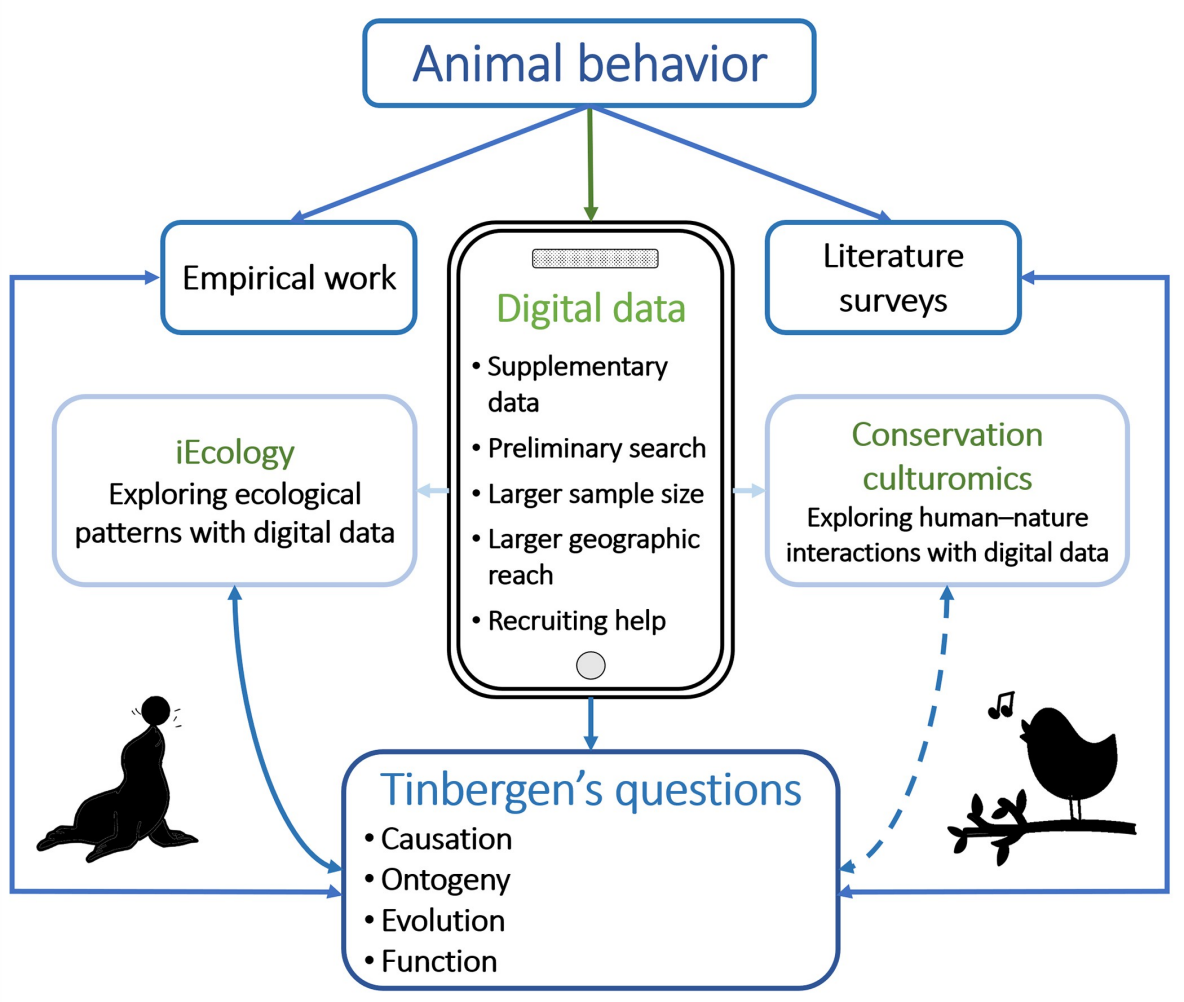
The potential contributions of digital data to understanding animal behavior. Traditionally, animal behavior has been studied mostly with empirical approaches and literature surveys. The addition of digital data enables us to explore ecological patterns (iEcology) and human–nature interactions (conservation culturomics). All of these approaches can help address Tinbergen’s questions of behavior. In return, Tinbergen’s questions help direct and shape research questions, experimental setups, and data collection. Conservation culturomics infers human behavior related to nature and is thus represented with a dashed arrow. Icons taken from https://openclipart.org/.

In the mid-20th century, Nikko Tinbergen created a foundational framework for the integrative study of animal behavior [12,13] by posing 4 interlinked questions regarding the 4 main axes of behavior: *causation*, the mechanistic basis of behavior; *ontogeny*, its development throughout an individual’s lifetime; *evolution*, its changes over an evolutionary time scale; and *function*, its adaptive value and current utility. Answering Tinbergen’s questions can be hindered by many research challenges including, but not restricted to, limited funds, time, accessibility, and sample sizes. In such cases, readily available data from various online platforms such as citizen science databases or social media platforms (for example, YouTube, Facebook, or Flickr) can prove to be a powerful and complementary tool to traditional methods involving observations and experiments (Fig 2) [4,7]. Furthermore, social media platforms, similar to citizen science platforms, can also provide bridges between scientists and nature enthusiasts (as well as the general public) that can be harnessed to help create and review large data sets. This, in turn, can also encourage people to reconnect with nature and promote biodiversity conservation [14].

Here, we propose that digital data, especially from social media platforms, can be used to answer questions beyond species distribution and occurrence to advance the field of animal behavior (Fig 2). While keeping in mind that Tinbergen’s questions are interlinked and complementary to each other, we explore each question separately, highlighting both opportunities and challenges in using digital data to answer them. We further highlight the increased relevance of Tinbergen’s questions to biodiversity conservation. We showcase instances where digital data has already been used to study animal behavior (Fig 1 and S1 Table) and suggest possible avenues for further research incorporating digital data to address fundamental and applied behavioral issues.

### Causation

Studies dealing with causation try to understand what causes a behavior to be performed. When combined with remotely sensed, freely available data, digital data sources can be used to explore the external mechanisms underlying a behavioral trait. For example, Cabello-Vergel and colleagues [15] combined data on the thermoregulatory behavior of individual storks (Ciconiidae) from georeferenced images and videos found at the Macaulay Library repository (https://www.macaulaylibrary.org) with remotely sensed microclimate data. They investigated the determinants of “urohidrosis” (excreting onto the legs as a form of evaporative cooling) in 19 stork species. They found that high heat loads (high temperature, humidity, and solar radiation, and low wind speed) promoted the use of urohidrosis and thus evaporative heat loss. In the face of global climate change, exploring shifts in mechanisms of control with microclimate data can inform us about mechanisms of adaptation to changing environments and provide profound insights facilitating future conservation efforts.

The study of social learning and the emergence of novel and innovative behaviors in relation to environmental conditions could particularly benefit from digital data sources because people often record surprising or unexpected animal behaviors [7]. For example, data from multiple digital data sources revealed that 10 out of the 16 world’s terrestrial hermit crab species (*Coenobitidae*) widely use artificial shells, predominantly plastic caps, but also pieces of glass or metal [16]. This novel behavior may be driven by decreased availability of gastropod shells, sexual signaling, lightness of artificial shells, odor cues, and/or camouflage in a polluted environment. Together with controlled preferences experiments and/or records of pollution levels and other environmental conditions, we can address the underlying mechanisms of this behavior, which may ultimately influence the evolutionary trajectory of the species. Other examples include YouTube videos that have been used to describe horses opening doors and gate mechanisms [17] or investigate death-related behavioral responses in Asian elephants (*Elephas maximus*) such as carrying dead calves [18]. Understanding why and when these rare behaviors occur may not be possible without such online records.

In 2022, Møller and Xia [19] showed that bird species recorded on YouTube videos feeding directly from people’s hands also presented more innovative behaviors, had a higher rate of introduction success, and greater urban tolerance than species not recorded displaying such behavior. This demonstrates the connections between Tinbergen’s questions and highlights that an individual’s (or species) ability to respond behaviorally to external conditions may also rely on its evolutionary history and affects its chances of survival. It further shows that the fields of urban ecology and invasion biology can greatly benefit from integrating these novel digital data sources. For example, with most of the global human population living in cities and the omnipresence of online social platforms, digital data can make global multi-city comparisons of urbanization effects on species behavior feasible. Moreover, human activity can be easily tracked using mobility reports provided by Google (https://www.google.com/covid19/mobility/) and Apple (https://covid19.apple.com/mobility). These can provide a high-resolution understanding of where and when humans are active and how they can play an important role in shaping animal behavior. Such knowledge can help enhance studies of antipredator behavior and wildlife tolerance, as it was used to study the consequences of the COVID-19 pandemic lockdowns [20,21]. Likewise, documenting first arrivals and monitoring the spread of invasive species, their behavior, and interactions with native species can become more efficient by incorporating digital data from online repositories [22].

We acknowledge that digital sources alone cannot offer many insights into internal mechanisms of behavior, such as hunger state or past experience (exceptions may include behaviors that are influenced by temperature, which may be inferred if the data are georeferenced and time stamped). Studying proximate physiological mechanisms often requires extensive field and laboratory experiments. However, addressing what mechanisms drive behavior in terms of changes in the external stimulus (social and physical environment) could greatly benefit from the copious number of available images and videos online. This is particularly true considering current and future global environmental challenges.

### Ontogeny

Digital data in the forms of images, audio, videos, and live-streaming videos can also be used to study and quantify different behavioral shifts in individuals over their lifetimes. For example, using online-sourced photographs, Naude and colleagues [23] showed that adult martial eagles (*Polemaetus bellicosus*) preyed more on birds than juveniles and subadults, which preferred less agile reptiles and mammals. They attributed this pattern to an improvement in hunting skills with age. Another study found evidence for “ontogenetic deepening”—the phenomenon that older and larger fish are found in deeper water, whereas younger and smaller fish stay in shallower water—in dusky groupers (*Epinephelus marginatus*) using YouTube videos of recreational fishers [24]. Exploring videos over several years, they further showed that fishing depth did not change over time and thus suggested that this ontogenetic deepening may not be solely driven by changes in harvesting pressure. Combining acoustic recordings from various sources (field recordings, a museum sound library, and citizen science records), Riós-Chelén and colleagues [25] found that birds can adapt their songs to environmental acoustic conditions. The fact that songbirds (known as oscines), who learn their songs, showed stronger associations between environmental noise and song modifications than other closely related bird species with innate songs (suboscines) indicates the involvement of ontogenetic processes in this adjustment.

Other studies can use similar approaches to further explore ontogenetic changes in different species’ hunting skills, aggressiveness, mating rituals, and parental care, with or without complementing intensive fieldwork (see S1 Table). Exploring such changes in behavior in response to anthropogenic environmental changes worldwide can be of great importance for conservation science, urban ecology, and agroecology. For example, live-streaming videos of bird nests—which have become very common for many species and sites (e.g., https://camstreamer.com/blog/streaming-birds-with-an-eagle-eye and https://www.viewbirds.com/)—can provide rich information to study the development of nestling vocal signals, the learning of songs, or the establishment of siblings relationships, as well as differences in such behaviors as a function of the distance to urban areas, human disturbance level, or levels of noise or light pollution [26]. Nonetheless, similar to exploring *causation* mechanisms, answering questions related to ontogeny cannot solely rely on digital data sources since ontogenetic processes often involve studying individuals over time. Furthermore, developing a deep understanding of external factors affecting the development of behavior may also require well-designed controlled experiments, which can be more challenging to accomplish with currently available digital databases.

### Evolution

With images and videos from around the world spanning several decades available online, it is now possible to use digital data to explore intra- and interspecific traits and behaviors, as well as study their evolution in light of anthropogenic environmental changes. For example, using crowd-sourced images and videos, Mikula and colleagues [10] showed that predator–prey interactions between diurnal birds and bats, which were previously thought to be rare, have been commonly reported around the world (Fig 1). This indicates that diurnal bird predation might act as one of the drivers of the evolution of bat nocturnality. Similarly, using social media videos and phylogenetic modeling, Bastos and colleagues [27] showed that tool-using behavior in parrots is far more common than previously thought and that these new sources of data can be used to better understand the origin, evolution, and drivers of rare behaviors. In another example, Pearse and colleagues [28] were able to explore evolutionary patterns in bird song at a broad scale (in terms of pitch and complexity) using a large citizen science digital repository, combined with scientific data on bird biology, life history, and geographical distribution, and advanced machine learning techniques. Surprisingly, they showed that suboscine and oscine birds have similar song complexity. They further noted that using Artificial intelligence (AI) tools to help analyze citizen science data can further facilitate research on bird song evolution. However, such tools may also have limitations and need to be routinely validated and assessed.

The fact that digital repositories can potentially hold decades-old data allows retrospective explorations of data collected long before the research has commenced. For example, the COVID-19 pandemic highlighted the importance and usefulness of citizen science data sets, as past records could be compared with records under the novel environmental settings created by the pandemic [21]. Similar data sets may be obtained from various social media platforms that are far more popular than citizen science platforms, both in volume and in geographic coverage. For example, there are 3 million iNaturalist users (https://www.inaturalist.org/stats) compared with 300 million X (formerly Twitter) users (https://www.statista.com/statistics/303681/twitter-users-worldwide/). While most of the content on X would probably be irrelevant for ecology and conservation, the potential to reach and engage new audiences, and access diverse data could be valuable. Using these novel data sources can further facilitate large spatial scale explorations of evolutionary changes in animal behavior. It may also help researchers to better plan and choose field sites before embarking on intensive fieldwork.

Many aspects of the evolution of animal behavior are challenging to document directly because numerous phenotypic traits co-evolve over large spatial and phylogenetic scales, making comparative studies useful. For example, body coloration may be an important factor in answering fundamental questions in behavioral ecology that provides insights into local behavioral adaptations [29,30]. Online image repositories have already been used to document geographical and phylogenetic variation in color patterns in birds and mammals, including color polymorphism [31], mutations [32], and variation in the morphology of color strips and patches [33]. In addition to readily available data, people can be encouraged to upload their images, videos, and sound recordings for specific studies through citizen science platforms [34] or social media platforms [35]. Spatial data on the phenotypic distributions are often collected via field observations and inspection of voucher specimens.

We envision that online images, videos, and acoustic recordings may provide a rich resource of information on large-scale variation in many phenotypic traits closely linked to animal behavior, such as nest morphology in fish and birds, or the size and shape of ornaments and armaments (e.g., antlers in deer or bony spurs in birds). Yet, we must acknowledge the limitations of using digital data to answer questions of an evolutionary nature that require some genomic knowledge. Still, the sheer volume of digital data and the ability to compare data of many species and populations inhabiting different areas and environments can provide valuable information for the processes and mechanisms involved in evolutionary adaptation and speciation.

### Function

Answering function-related questions—how a behavior increases one’s fitness through survival and reproduction—can also gain much from using digital data. With the ubiquity of the Internet, we can explore external drivers of current utility and sexual selection regarding behavioral contributions to overall fitness. These may include intra- and interspecific interactions, migratory patterns, predation risk, and mating rituals. For example, using live-streaming underwater cameras, Coleman and Burge [36] showed a higher association between sand tiger sharks (*Carcharias taurus*) and round scads (*Decapterus punctatus*) in the presence of scad mesopredators, which enhances foraging opportunities for sand tiger sharks and reduces predation risk for the scads. Such behaviorally mediated indirect interactions may have far-reaching implications for trophic interactions, including predator and prey strategies. Studies like this highlight the potential of these novel data and technologies in ecological research.

Digital data can be further used to study the timing of biological processes (i.e., phenology) in animals and how these are being affected by external cues such as climate change, land use changes, or human disturbance. For example, using Wikipedia page views, Mittermeier and colleagues [37] tracked seasonal migration patterns in sockeye salmon (*Oncorhynchus nerka*) and Atlantic salmon (*Salmo salar*). Atsumi and Koizumi [38] used X (formerly Twitter) and Google Images to explore spatial variations in breeding timing in Japanese dace fish (*Tribolodon hakonensis*) and how they may have been affected by climate change. Combined with data on breeding success or the costs of not adjusting breeding timing, these studies could greatly advance function-related research. Given the ongoing global environmental change, such explorations can be invaluable to understanding how these changes impact various species in terms of range shifts and/or expansions. Again, digital data has limits, and complementing it with traditional methods may be required to accurately assess the fitness value of a behavior.

### The challenges and limitations of using digital data to study animal behavior

Addressing questions related to any of Tinbergen’s 4 levels of analysis is challenging. While digital data and approaches can greatly advance the fields of behavioral ecology and conservation behavior, these data sources and tools currently cannot replace empirical work and field studies. We acknowledge the limitations of digital data, particularly in answering questions related to internal mechanisms such as endocrine or neural control of behavior. Available digital data may not provide reliable information on an individual’s physiological state, its developmental history, or its reproductive state. Nonetheless, digital data sources can provide new opportunities to explore many aspects of Tinbergen’s 4 questions in a noninvasive way and without manipulation of free-living animals, thus solving underlying ethical and welfare issues associated with the use of animals in research [39]. It is important to note, however, that digital data research also raises ethical questions and should follow rules to avoid disruption to the focal animal(s), the animals’ population, or the wider ecosystem. Viewing digital data as complementary to more traditional sources of data may be very useful. Moreover, in some areas traditional data sources are lacking, and so adequately reliable digital data may be the best source of behavioral data available. Nevertheless, we must consider the biases, technical challenges, and ethical concerns associated with digital data.

First, data sets obtained from online platforms—particularly ones provided by the general public—have an inherited bias linked to Internet coverage and use such that different regions of the world are not equally represented in digital records. Similarly, different sectors of society based on, for example, ethnicity, language, socioeconomic status, and education level, are currently not equally represented in the digital realm, complicating research on human–nature interactions using digital data.

Second, only a fraction of the global biodiversity is digitally recorded and has an online presence [1,40]. This limits the number of species that can be explored using digital data sets and leads to an uneven sampling effort across different taxa and clades. Such biases, for example, towards charismatic or larger-bodied species, are widespread and well known from more traditional approaches of scientific research [41], but may be exacerbated using data from social media. Furthermore, this limitation of unequal human interest goes beyond which species are predominantly documented, but also to which behaviors are recorded. Such human preferences and biases, and how they may differ across cultures, may compromise analyses and conclusions if not properly accounted for [42]. Furthermore, search algorithms of search engines like Google or platform-internal ones may also introduce biases affecting the results returned.

The lack of rigorous collection protocols across various digital platforms, especially in light of the complexity and variety of animal behavior, makes applying digital data sources in behavioral ecology research even more challenging. For example, in exploring bird plumage color aberrations using various digital sources (Google Images and several local platforms devoted to bird watching and photography), Zbyryt and colleagues [32] highlighted how digital sources and public participation can advance our understanding of less-studied natural phenomena. They showed that color aberrations are more prevalent in urban, larger, and sedentary birds. However, the nature of the input data prevented them from concluding whether these patterns were biologically driven or resulted from inherent biases in their data set where people more easily spot and report large sedentary birds in human settlements. Thus, it is essential to address these and other biases and limitations to understand when and where it is appropriate to use various digital data sources. As a start, combining data from novel digital sources—such as various social media platforms and Google Images—with more rigorous scientific data sets, dedicated fieldwork, or literature surveys, can help validate digital sources and ensure meaningful results. Another approach is creating well-designed question-first citizen science data sets in which researchers recruit and train citizen scientists to collect dedicated data to answer specific questions [43].

When exploring user-generated content—for example, videos uploaded on social media platforms—we must also consider legal and ethical aspects such as data protection and privacy [44]. In order to minimize the risk of misusing sensitive data (e.g., IP address, localization details, or user name), we advocate for establishing and following protocols for data protection [44]. It is also important to note that many social media recordings may be associated with unintentional or even intentional disturbances and harmful actions towards the animal being recorded [5,45], raising ethical concerns as well as questions of interpretation and relevance. Even if individuals are not directly harmed, the context under which data were recorded (e.g., Were domestic animals like dogs present? Did the humans feed the animals before filming?) is not always known, and this may have substantial impacts on the recorded behavior [17]. Such human disturbances, combined with partial recording and suboptimal recording quality, necessitate extensive filtering processes and the implementing of clear protocols for the inclusion of records. Furthermore, it may limit the use of digital data sources in certain explorations [7]. While we encourage sharing nature observations online, we strongly discourage any actions that could harm the animals and the environment in the process. By contrast, recording people’s negative interactions with nature can potentially be helpful for both legal and conservation interventions, as well as for related research.

Finally, while these readily available data sets are relatively easy to obtain, using them requires programming skills, computational power, and storage capacity, among other things [46]. Accessing various platforms may further require data-sharing agreements, proprietary companies opening their data sets for researchers, and consistency in how data is managed [47]. Once obtained, data filtering and cleaning processes and analysis would further require advanced technological tools, such as machine learning methods and machine vision models. Such filtering process should also consider for example AI-generated content and ensure only reliable data are used. Post-analysis challenges may include repeatability and reproducibility [4,48] as data may not be archived on different platforms, and downloading and sharing all records may face legal issues (copyrights), as well as storage space limitations. While some of these aspects are beyond our control, keeping clear records of protocols, versions, and codes, as well as publishing metadata and when possible raw data, could increase transparency and help address some limitations [4].

## Conclusions and future outlook

The use of digital data in ecological and evolutionary research on animal behavior has emerged as a promising approach to enhance traditional data sources and overcome several constraints such as lack of time, accessibility, and financial resources. Digital data enables researchers to conduct retrospective analysis and comparisons across various temporal, spatial, and taxonomic scales, providing a potentially vast data set to explore. Moreover, as Internet use continues to grow and new digital platforms emerge, more data will become available, offering further opportunities to advance both basic and applied studies in behavioral ecology. The use of digital data in behavioral ecology is rapidly increasing and will potentially unveil larger data sets and larger audiences than existing citizen science platforms [35,49]. These new databases will enable researchers to ask basic and novel questions and study animal behavior with greater depth and scope. Furthermore, by leveraging social media data created by individuals, researchers can advance knowledge on animal (including human) behavior, promote public engagement with nature, and enhance present and future conservation efforts.

In addition to using data already uploaded to the Internet, scientists can encourage people to upload data containing species or areas of interest for their study. Researchers can also recruit people to help filter, score, or tag data collected online as on the Zooniverse platform (https://www.zooniverse.org/), with the ultimate goal of involving the public in biodiversity conservation and science and facilitating the processing of big data. With advances in AI models, such collection and classification of data can be made automatically (fully or semi), based on taxonomic group or the location where the observation was recorded. This will enhance the ability of researchers to incorporate publicly available data in their studies. For example, using machine learning approaches, Pardo and Wittemyer [50] were able to find a name-like calling behavior in African savannah elephants (*Loxodonta africana*). However, limited by their sample size, they were not able to isolate and encode specific “name” sounds. Social media recording of tourists in those areas could potentially help in future research.

With the increasing global environmental challenges linked to biodiversity loss and climate change, digital resources are invaluable sources of data, especially in time-sensitive cases. Behavioral aspects such as interspecific interactions or behavioral flexibility are missing from many large-scale analyses and predictions of future species responses to human-driven environmental changes [51,52]. Digital data can greatly improve our ability to successfully integrate such behavioral dimensions into spatial modeling of abiotic changes and help us produce more realistic estimates of future risks and potential species distributions [52]. Taken together, such studies can help us develop a rich understanding of behavior based on the Tinbergen framework.

From an applied perspective, the field of conservation behavior [53] can benefit substantially from digital data sources too. Online images and video repositories can help conservation scientists and managers better understand anthropogenic impacts on animal behavior, identify behavioral indicators of changes to the species’ environment, highlight potential human–wildlife conflicts, and design and implement behavior-sensitive management [54]. With the great advancements in AI and machine learning and the increased availability of big data, we expect that more behavioral ecologists and conservation scientists will start incorporating digital-based data sources and approaches alongside their field and empirical work.

## Supporting information

S1 Table Examples of publications utilizing digital data for behavioral ecology divided into their potential contribution to understanding animal behavior according to Tinbergen’s 4 questions. (DOCX)
