# Supplementary material for: Leveraging social media and other online data to study animal behavior
Source: PLoS Biol. 2024 Aug 29;22(8):e3002793. doi: 10.1371/journal.pbio.3002793 (PMC11389916; doi:10.1371/journal.pbio.3002793)
Supplement: S1 Table — (DOCX) [file pbio.3002793.s001.docx]

**S1 Table.** **Examples of publications utilizing digital data for behavioral ecology divided into their potential contribution to understanding animal behavior according to Tinbergen’s four questions.**

Each publication was scored based on its relevance to each of Tinbergen’s questions of behavior with 1 signifying the study has little to do with this question, 2 meaning the study’s data could potentially be used to explore questions related to this question, 3 meaning the study is not focused on this question but can contribute to understanding it, and 4 meaning the study directly addresses questions related to this behavioral aspect. The table also shows the data sources used by each publication.

|  | **Papers** | **Causation** | **Ontogeny** | **Evolution** | **Function** | **Data sources** |
| --- | --- | --- | --- | --- | --- | --- |
| 1 | Angarita-Sierra T, Montaño-Londoño LF, Bravo-Vega CA. ID please: Evaluating the utility of Facebook as a source of data for snake research and conservation. An Acad Bras Cienc. 2022;94:1–25. | 2 | 1 | 2 | 2 | Novel digital sources: Facebook |
| 2 | Atsumi K, Koizumi I. Web image search revealed large-scale variations in breeding season and nuptial coloration in a mutually ornamented fish, Tribolodon hakonensis. Ecol Res. 2017;32(4):567–78. | 3 | 1 | 4 | 2 | Novel digital sources: Google Images, Twitter |
| 3 | Boydston EE, Abelson ES, Kazanjian A, Blumstein DT. Canid vs. canid: insights into coyote-dog encounters from social media. Human-Wildlife Interact. 2018;12(2):233–42. | 3 | 2 | 1 | 3 | Novel digital sources: YouTube |
| 4 | Cabello-Vergel J, Soriano-Redondo A, Villegas A, Masero JA, Guzmán JMS, Gutiérrez JS. Urohidrosis as an overlooked cooling mechanism in long-legged birds. Sci Rep [Internet]. 2021;11(1):1–11. Available from: https://doi.org/10.1038/s41598-021-99296-8 | 4 | 1 | 4 | 3 | Scientific databases: Macaulay Library repository |
| 5 | Coleman NC, Burge EJ. Association behavior between sand tiger sharks and round scad is driven by mesopredators. PeerJ. 2021;9:1–30. | 3 | 1 | 2 | 4 | Novel digital sources: SharkCam live-stream camera |
| 6 | Drury JP, Barnes M, Finneran AE, Harris M, Grether GF. Continent-scale phenotype mapping using citizen scientists’ photographs. Ecography (Cop). 2019;42(8):1436–45. | 1 | 1 | 1 | 2 | Citizen science datasets: iNaturalist |
| 7 | Dylewski ?, Mikula P, Tryjanowski P, Morelli F, Yosef R. Social media and scientific research are complementary—YouTube and shrikes as a case study. Sci Nat. 2017;104(5–6):1–7. | 2 | 1 | 2 | 1 | Novel digital sources: YouTube |
| 8 | Fuller LN, Parsons GR. A Note on Associations Observed between Sharks and Teleosts. Southeast Nat. 2019;18(3):489–98. | 2 | 2 | 2 | 3 | Novel digital sources: Google Images |
| 9 | Gutiérrez JS, Soriano-Redondo A. Laterality in foraging phalaropes promotes phenotypically assorted groups. Behav Ecol. 2021;31(6):1429–35. | 4 | 2 | 3 | 2 | Scientific databases and Noverl digital sources: Macaulay Library, BBC Motion Gallery, YouTube, Vimeo, Flickr, Gettyimages |
| 10 | Hernandez M, Masonick P, Weirauch C. Crowdsourced online images provide insights into predator-prey interactions of putative natural enemies. Food Webs [Internet]. 2019;21:e00126. Available from: https://doi.org/10.1016/j.fooweb.2019.e00126 | 1 | 1 | 2 | 2 | Citizen science datasets and Noverl digital sources: Flickr, Google, iSpot Nature, BugGuide, NatureWatch |
| 11 | Jagiello ZA, Dyderski MK, Dylewski ?. What can we learn about the behaviour of red and grey squirrels from YouTube? Ecol Inform. 2019;51(January):52–60. | 2 | 2 | 2 | 3 | Novel digital sources: YouTube |
| 12 | Jagiello Z, Dylewski ?, Szulkin M. The plastic homes of hermit crabs in the Anthropocene. Sci Total Environ. 2024;913(August 2023). | 4 | 1 | 4 | 2 | Citizen science datasets and Noverl digital sources: iNaturalist, Flickr, Google Images, YouTube, Alamy |
| 13 | Krueger K, Esch L, Byrne R. Animal behaviour in a human world: A crowdsourcing study on horses that open door and gate mechanisms. PLoS One. 2018;14(6):1–20. | 2 | 4 | 2 | 2 | Citizen science datasets: dedicated project website |
| 14 | Leitão ATTS, Alves MD d. O, dos Santos JCP, Bezerra B. Instagram as a data source for sea turtle surveys in shipwrecks in Brazil. Anim Conserv. 2022;25(6):736–47. | 2 | 2 | 1 | 2 | Novel digital sources: Instagram |
| 15 | Maritz RA, Maritz B. Sharing for science: High-resolution trophic interactions revealed rapidly by social media. PeerJ. 2020;2020(7). | 2 | 1 | 2 | 2 | Novel digital sources: Facebook |
| 16 | Mikula P, Hadrava J, Albrecht T, Tryjanowski P. Large-scale assessment of commensalistic-mutualistic associations between African birds and herbivorous mammals using internet photos. PeerJ. 2018;2018(3):1–23. | 2 | 1 | 3 | 2 | Novel digital sources: Google Images |
| 17 | Mikula P, Morelli F, Lu?an RK, Jones DN, Tryjanowski P. Bats as prey of diurnal birds: SA global perspective. Mamm Rev. 2016;46(3):160–74. | 1 | 1 | 2 | 1 | Novel digital sources: Google, Google Books, Google Images, Flickr, YouTube |
| 18 | Miranda EBP, Ribeiro-Jr. RP, Strüssmann C. The ecology of human-anaconda conflict: a study using internet videos. Trop Conserv Sci. 2016;9(1):43–77. | 2 | 1 | 1 | 2 | Novel digital sources: Google Videos |
| 19 | Møller AP, Xia C. The ecological significance of birds feeding from the hand of humans. Sci Rep. 2020;10(1):1–5. | 2 | 1 | 4 | 1 | Novel digital sources: YouTube |
| 20 | Mori E, Di Bari P, Coraglia M. Interference between roe deer and Northern chamois in the Italian Alps: are Facebook groups effective data sources? Ethol Ecol Evol [Internet]. 2018;30(3):277–84. Available from: https://doi.org/10.1080/03949370.2017.1354922 | 3 | 1 | 1 | 3 | Novel digital sources: Facebook |
| 21 | Nascimento LS, Noernberg MA, Bleninger TB, Hausen V, Pozo A, Camargo LS, et al. Social media in service of marine ecology: new observations of the ghost crab Ocypode quadrata (Fabricius, 1787) scavenging on Portuguese man-of-war Physalia physalis (Linnaeus, 1758). Aquat Ecol. 2022;56(3):859–64. | 1 | 1 | 2 | 2 | Novel digital sources: Instagram |
| 22 | Naude VN, Smyth LK, Weideman EA, Krochuk BA, Amar A. Using web-sourced photography to explore the diet of a declining African raptor, the Martial Eagle (Polemaetus bellicosus). Condor. 2019;121(1):1–9. | 2 | 4 | 2 | 2 | Novel digital sources: Google Images, Tineye |
| 23 | O'Reilly C, Analuddin K, Kelly DJ, Harte N. Measuring vocal difference in bird population pairs. 2018;1671:1658–71. Available from: http://dx.doi.org/10.1121/1.5027244 | 2 | 1 | 4 | 2 | Novel digital sources: YouTube, Facebook |
| 24 | Pace DS, Giacomini G, Campana I, Paraboschi M, Pellegrino G, Silvestri M, et al. An integrated approach for cetacean knowledge and conservation in the central Mediterranean Sea using research and social media data sources. Aquat Conserv Mar Freshw Ecosyst. 2019;29(8):1302–23. | 2 | 1 | 2 | 3 | Citizen science datasets: xeno-canto database |
| 25 | Pearse WD, Morales-Castilla I, James LS, Farrell M, Boivin F, Davies TJ. Global macroevolution and macroecology of passerine song. Evolution (N Y). 2018;72(4):944–60. | 4 | 1 | 4 | 2 | Novel digital sources: YouTube |
| 26 | Pokharel SS, Sharma N, Sukumar R. Viewing the rare through public lenses: Insights into dead calf carrying and other thanatological responses in Asian elephants using YouTube videos. R Soc Open Sci. 2022;9(5). | 2 | 1 | 2 | 2 | Novel digital sources: YouTube |
| 27 | Ri´os-Chele´n AA, Salaberria C, Barbosa I, Macias Garcia C, Gil D. The learning advantage?: bird species that learn their song show a tighter adjustment of song to noisy environments than those that do not learn. J Evol Biol. 2012;25:2171–80. | 2 | 4 | 3 | 4 | Novel digital sources: Instagram |
| 28 | Sbragaglia V, Coco S, Correia RA, Coll M, Arlinghaus R. Analyzing publicly available videos about recreational fishing reveals key ecological and social insights: A case study about groupers in the Mediterranean Sea. Sci Total Environ [Internet]. 2021;765:142672. Available from: https://doi.org/10.1016/j.scitotenv.2020.142672 | 2 | 4 | 1 | 1 | Scientific databases and Citizen science datasets: Macaulay Library repository, iNaturalist |
| 29 | Sullivan M, Robinson S, Littnan C. Social media as a data resource for #monkseal conservation. PLoS One. 2019;14(10):1–11. | 2 | 1 | 1 | 2 | Novel digital sources: Google Images, local forums and websites dedicated to ornithology, birdwatching and bird photography |
| 30 | Vrettos M, Reynolds C, Amar A. Malar stripe size and prominence in peregrine falcons vary positively with solar radiation: Support for the solar glare hypothesis. Biol Lett. 2021;17(6):1–7. | 1 | 1 | 1 | 4 | Citizen science datasets: xeno-canto database |
| 31 | Zbyryt A, Mikula P, Ciach M, Morelli F, Tryjanowski P. A large-scale survey of bird plumage colour aberrations reveals a collection bias in Internet-mined photographs. Ibis (Lond 1859). 2021;163(2):566–78. | 1 | 1 | 4 | 1 | Scientific databases and Citizen science datasets: Macaulay Sound Library, the sound library of the Museo Nacional de Ciencias Naturales, Xeno-canto database |
| 32 | Bastos APM, Claessens S, Nelson XJ, Welch D, Atkinson QD, Taylor AH. Crowdsourcing and phylogenetic modelling reveal parrot tool use is not rare Amalia. bioRxiv. 2023; | 2 | 1 | 4 | 2 | Novel digital sources: YouTube |
| 33 | Hutchinson JR, Pringle E V. Footfall patterns and stride parameters of Common hippopotamus (Hippopotamus amphibius) on land. PeerJ. 2024;12(7):1–15. | 2 | 2 | 2 | 1 | Novel digital sources: YouTube, BBC Natural earth clip |
| 34 | Sharp TR, Garshelis DL, Larson W. A most aggressive bear: Safari videos document sloth bear defense against tiger predation. Ecol Evol. 2024;14(7):1–22. | 3 | 1 | 3 | 3 | Novel digital sources: Google, YouTube, Yahoo, and Bing |
